# Stress Fracture of the Pubic Ramus With Associated Osteitis Pubis in a Young Football Player: A Case Report

**DOI:** 10.7759/cureus.109993

**Published:** 2026-05-31

**Authors:** Abdelrahman Akkoumi, Reda Bahij, Ali Haidar, Omar Aguenaou, Mohammed-Reda Fekhaoui

**Affiliations:** 1 Department of Trauma and Orthopaedic Surgery, Ibn Sina University Hospital, Faculty of Medicine, Mohammed V University of Rabat, Rabat, MAR; 2 Department of Radiology, Ibn Sina University Hospital, Faculty of Medicine, Mohammed V University of Rabat, Rabat, MAR

**Keywords:** football player, mri, osteitis pubis, pelvic pain, pubic symphysis, stress fracture

## Abstract

We report the case of a 21-year-old male semi-professional football player presenting with a one-month history of progressive inguino-pubic pain without a history of acute trauma. Clinical examination revealed point tenderness over the pubic symphysis and pain on resisted adductor contraction. Standard pelvic radiographs were unremarkable. Magnetic resonance imaging (MRI) demonstrated a stress fracture of the left inferior pubic ramus with associated stress reaction of the pubic body, osteitis pubis, and oedema at the left adductor insertion.

Conservative management comprising immediate cessation of sporting activity, progressive protected weight bearing, analgesia, and structured physiotherapy resulted in complete resolution of symptoms and a gradual return to competitive football without complications.

Stress fractures of the pubic symphysis should be considered in the differential diagnosis of groin pain in athletes. MRI is the imaging modality of choice for early diagnosis and characterisation of associated soft-tissue lesions. Conservative treatment with structured rehabilitation yields favourable outcomes.

## Introduction

Stress fractures result from an imbalance between the repetitive mechanical loads applied to bone and its capacity for adaptive remodelling. While most common in the lower limb, pelvic stress fractures remain a rare but increasingly recognised entity in competitive athletes [1,2]. Involvement of the pubic symphysis and pubic rami is particularly uncommon and is primarily described in sports requiring repetitive unilateral loading, explosive acceleration, and abrupt direction changes - most notably football (soccer) - with fractures of the pubic rami representing the predominant osseous injury pattern in this context [3,4].

Pelvic stress fractures account for a minority of all stress fractures in athletes, with pubic rami involvement representing a particularly uncommon subtype described predominantly in football players and distance runners [1,2].

The diagnosis is frequently delayed because the symptomatology closely overlaps with more common conditions including osteitis pubis, adductor tendinopathy, and inguinal hernia. Plain radiography is insensitive in the early phase of injury, and MRI has emerged as the imaging modality of choice for early detection, allowing identification of bone marrow oedema, fracture lines, and associated soft-tissue pathology [3,5].

We report a case of stress fracture of the pubic ramus with associated osteitis pubis in a young competitive footballer, with the aim of highlighting the clinical features, imaging findings, and conservative management of this uncommon but important diagnosis.

## Case presentation

A 21-year-old male semi-professional football player with no significant past medical history presented with a one-month history of progressive inguino-pubic pain. He reported no history of acute trauma. The pain had initially been triggered exclusively by sporting activity - running, kicking, and direction changes - but had subsequently become present during activities of daily living. The patient reported a significant recent increase in training load.

Clinical examination

Clinical examination revealed point tenderness on palpation of the pubic symphysis, pain on resisted adductor contraction, discomfort on single-leg stance, and preserved hip range of motion with pain only at end range; no neurological deficit was identified.

Imaging

Plain Radiography

Anteroposterior pelvic radiography demonstrated preserved alignment of the pelvic ring and normal-appearing hip joints. No definite fracture line or displacement was visible at the pubic symphysis or pubic rami (Figure 1), illustrating the limited sensitivity of plain radiography in the early diagnosis of pelvic stress fractures.

**Figure 1:**
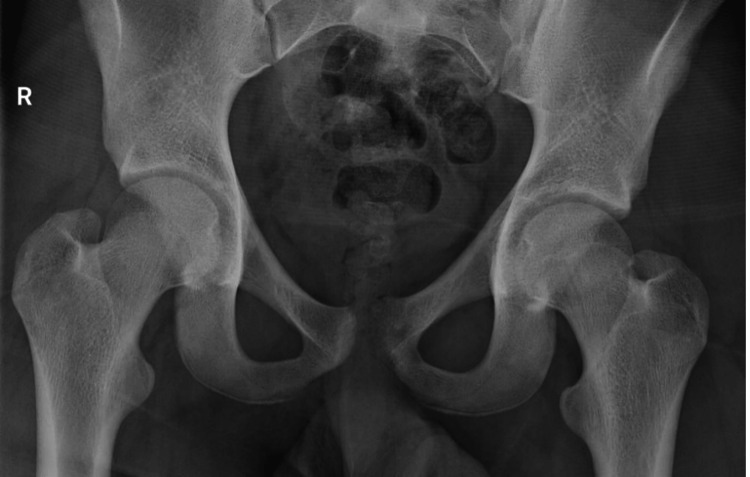
Anteroposterior pelvic radiograph demonstrating preserved pelvic ring alignment and normal hip joints. No fracture line or displacement is visible at the pubic symphysis, highlighting the low sensitivity of plain radiography in early pelvic stress fractures.

Radiographic interpretation was performed by the treating orthopaedic team, and findings were subsequently reviewed and confirmed by a staff radiologist.

Magnetic Resonance Imaging

Pelvic MRI (Figures 2-4) revealed a stress reaction with a non-displaced fracture line of the left inferior pubic ramus, best visualised on fat-suppressed T2-weighted sequences, as well as a stress reaction of the pubic body. Features consistent with osteitis pubis were noted, with high signal intensity at the symphyseal margins on fat-suppressed T2-weighted sequences, along with moderate oedema at the left adductor insertion. No abnormality of the rectus abdominis aponeurosis or acute significant soft-tissue injury was identified.

**Figure 2:**
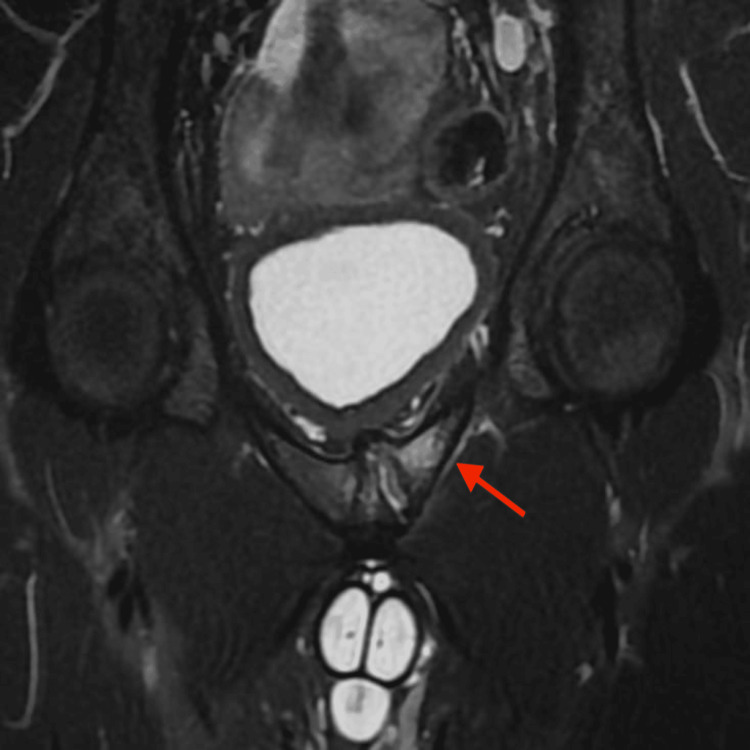
Pelvic MRI, fat-suppressed T2-weighted sequence, demonstrating hyperintense signal at the margins of the pubic symphysis consistent with stress-related bone marrow oedema (red arrow).

**Figure 3:**
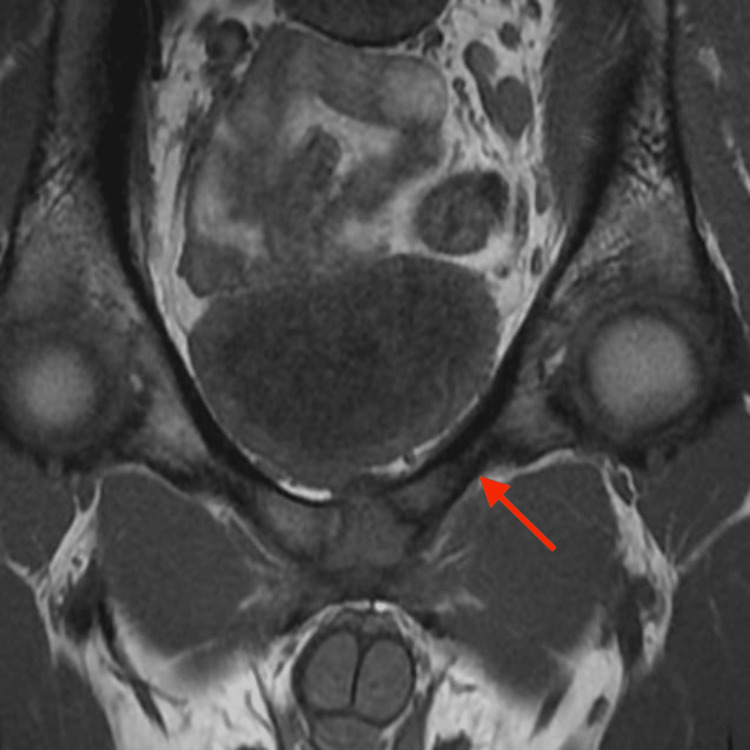
Pelvic MRI showing a fine hypointense intra-osseous line within the left inferior pubic ramus, compatible with a non-displaced stress fracture (red arrow).

**Figure 4:**
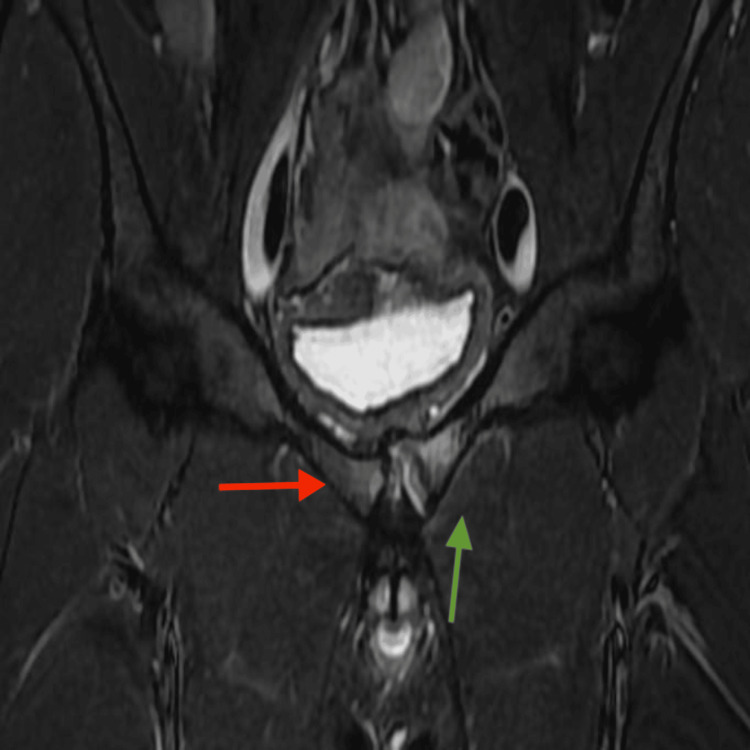
Pelvic MRI demonstrating oedema at the left adductor insertion (green arrow) with associated features of osteitis pubis (red arrow), findings frequently observed in pelvic overuse injuries in footballers.

Management and outcome

A conservative management strategy was adopted in three progressive phases.

Phase 1 (weeks one to three) included complete cessation of all sporting activity, protected weight-bearing with crutches as needed guided by pain, oral analgesics (nonsteroidal anti-inflammatory drugs (NSAIDs) and paracetamol), and passive physiotherapy including gentle range-of-motion and soft-tissue work targeting the adductors and hip flexors.

Phase 2 (weeks four to six) included progressive introduction of impact-free exercise including cycling and swimming, isometric core stabilisation exercises, and gradual adductor strengthening at sub-pain threshold.

Phase 3 (weeks seven to 20) included sport-specific progressive loading, including jogging, followed by running and cutting movements, with return to full training contingent on complete symptom resolution and painless single-leg stance. Return to competitive football was authorised upon complete clinical symptom resolution, painless resisted adductor contraction, and the ability to perform sport-specific movements without discomfort. 

The patient was followed for a total of 20 weeks. At final follow-up, complete resolution of inguino-pubic pain was documented, with full pain-free range of motion of the hip and painless resisted adductor contraction. At final follow-up, the patient reported a visual analogue scale (VAS) pain score of 0/10 at rest and during sporting activity. The patient returned to competitive football at week 10 of the rehabilitation programme, at his pre-injury level of performance, without complication. No follow-up imaging was performed prior to return to sport; the decision to return was based on clinical criteria alone, comprising complete symptom resolution, painless resisted adductor contraction, and full sport-specific movement tolerance.

## Discussion

Stress fractures of the pubic symphysis are rare but represent an important cause of inguinal and pelvic pain in athletes. They arise as a consequence of repetitive microtraumatic loading, driven by the opposing forces exerted by the adductor muscles and the anterior abdominal wall musculature on the pubic body and symphysis [6,7]. In footballers, the combination of repetitive kicking, acceleration, and lateral cutting movements creates cyclical shear and tensile stresses at the pubic symphysis that may exceed the threshold for stress fracture formation [4,8].

Clinically, pubic stress fractures are frequently misdiagnosed as osteitis pubis, adductor tendinopathy, or inguinal hernia, owing to the non-specific nature of inguino-pubic pain and the significant clinical overlap between these conditions [5,9]. A careful history identifying the absence of acute trauma, the presence of progressive activity-related pain, and a recent escalation in training load should alert the clinician to the possibility of a stress fracture.

Plain radiography has a well-documented low sensitivity for early stress fractures of the pelvis, with a negative result not excluding the diagnosis [3]. MRI is the imaging modality of choice, providing excellent soft-tissue contrast and the ability to identify bone marrow oedema - the earliest imaging hallmark of stress injury - as well as frank fracture lines, osteitis pubis, and adductor enthesopathy [5,7]. In our case, MRI identified a non-displaced fracture of the left inferior pubic ramus, bone marrow oedema of the pubic body, osteitis pubis, and adductor enthesopathy, none of which were apparent on plain radiography.

The present case illustrates the diagnostic challenge inherent to pelvic overuse injuries in footballers, where osteitis pubis, adductor enthesopathy, athletic pubalgia, and pubic stress fracture frequently co-exist or present with overlapping symptoms. The distinguishing feature of a stress fracture is the presence of a discrete fracture line or cortical irregularity on MRI - as demonstrated here within the left inferior pubic ramus - rather than the diffuse symphyseal signal change characteristic of isolated osteitis pubis. Athletic pubalgia, by contrast, is characterised by posterior inguinal wall weakness and abnormality of the rectus abdominis aponeurosis, which was absent in this case. Accurate MRI interpretation with attention to all pelvic structures is therefore essential to establish the precise diagnosis and guide appropriate management.

Several cases of pubic stress fractures in football players have been reported in the literature, though the injury remains uncommon compared to lower limb stress fractures. The majority of published cases involve male players in the second or third decade of life, consistent with our patient's profile. Reported cases typically present with progressive groin pain following a period of intensified training, with MRI demonstrating bone marrow oedema of the pubic body or rami and frequent co-existence of osteitis pubis and adductor enthesopathy - findings mirrored in the present case. Conservative management has been the standard approach in all non-displaced cases, with return to sport generally achieved within six to 12 weeks, consistent with our patient's favourable outcome [1,8,9].

Treatment of pubic stress fractures is conservative in the vast majority of cases, with surgical fixation reserved for displaced fractures or those failing prolonged conservative management. The cornerstone of treatment is relative rest and cessation of the offending activity, followed by a structured progressive rehabilitation programme targeting the core and hip stabilisers [1,10]. Return to sport is guided by clinical symptom resolution and imaging evidence of fracture healing, and typically occurs within six to 12 weeks in uncomplicated cases [8].

The present case is instructive in several respects. The diagnosis was delayed by one month due to initial non-specific presentation and the normal appearance of plain radiographs - a well-recognised pitfall in this condition. MRI established the definitive diagnosis and guided a targeted, non-operative management strategy that resulted in complete recovery. The association of osteitis pubis and adductor enthesopathy with the stress fracture in this case underscores the complex multi-structural nature of pelvic overuse injury in footballers and the importance of comprehensive MRI evaluation.

This report has several limitations inherent to the case report design. The absence of a validated functional outcome score - such as the Copenhagen Hip and Groin Outcome Score (HAGOS) or the Hip Outcome Score (HOS) - limits the objective assessment of recovery. No follow-up imaging was performed prior to return to sport, and the decision to return was based on clinical criteria alone. The single-patient design precludes generalisation of findings. Future prospective studies capturing pubic stress fractures in football athletes would provide higher-level evidence to guide management and define optimal return-to-sport criteria.

## Conclusions

Stress fracture of the pubic ramus with associated osteitis pubis is a rare but clinically significant cause of groin pain in athletes, particularly footballers. It should be included in the differential diagnosis of any athlete presenting with progressive inguino-pubic pain following an increase in training load, even in the absence of acute trauma. Plain radiography is insensitive for this diagnosis and should not be used to exclude it. MRI is the imaging modality of choice and is essential for early diagnosis, assessment of fracture extent, and identification of associated soft-tissue lesions. Conservative management with structured rehabilitation yields favourable outcomes, with a successful return to competitive sport.
